# Transparency and reproducibility: A step forward

**DOI:** 10.1002/hsr2.117

**Published:** 2019-03-07

**Authors:** Alejandro Montenegro‐Montero, Alberto L. García‐Basteiro

**Affiliations:** ^1^ John Wiley and Sons Hoboken New Jersey USA; ^2^ Amsterdam Institute for Global Health and Development (AIGHD) Amsterdam The Netherlands; ^3^ Barcelona Institute for Global Health (ISGlobal) Hospital Clinic‐Universitat de Barcelona Barcelona Spain; ^4^ Centro de Investigação em Saude de Manhiça (CISM) Maputo Mozambique

At *Health Science Reports*, we aim to publish scientifically sound and reproducible research. A key aspect of this is proper reporting and openness in all aspects of scientific communication. There is a growing demand for transparency, openness, and reproducibility in research, and several initiatives have been developed to try to address such needs.

In this editorial, we want to highlight some of the policies that the journal has implemented or that will implement in the coming year to support these efforts.

## 1 REPORTING

Reporting guidelines and standards are key to maximizing the quality and transparency of the research process and publication. Accurate and complete reporting enables readers to fully appraise research, to replicate the research where necessary, and crucially, use and interpret the research in an appropriate way.

Authors are currently encouraged to review the standards from the EQUATOR network (http://www.equator‐network.org/) and follow the relevant guidelines for the reporting of their specific study type (eg, STROBE for observational studies). For manuscripts submitted on or after March 1, 2019, and at manuscript submission, authors will need to confirm that they have reviewed the standards in the network, report whether any standards were relevant for their study, and, when appropriate, confirm that they have followed those standards in the manuscript. The completed checklists may be requested during the manuscript submission process and peer review.

## 2 AUTHORSHIP

Authorship implies responsibility and accountability for published work. We follow the International Committee of Medical Journal Editors (ICMJE) criteria for authorship. Moreover, and with the goal of providing a clear and transparent description of the contributions made by each author, we have implemented the Contributor Roles Taxonomy (CRediT), a controlled vocabulary of contributor roles which aims to improve accessibility and visibility of the range of contributions to published research outputs^1^. Implementation of this initiative has been seamless, and we will continue to request such statement, which we believe has numerous important and practical benefits.

In addition, and considering ICMJE guidelines, from January 1, 2019, we have been requesting authors to state whether the source(s) of support for the work or any other financial relationship, played any role in study design; collection, analysis, and interpretation of the data; writing of the report; or the decision to submit it for publication. Further, we have been requesting that one named author (eg, lead author or the manuscript guarantor) and no more than two authors indicate that they had full access to all of the data and that they take complete responsibility for its integrity and for the accuracy of its analysis. Similarly, a statement certifying that all authors have read and approved the final version of the manuscript, which is already being requested as part of the submission process, will now be published with the manuscript.

Lastly, articles submitted from March 1, 2019 onwards will need to include a declaration of transparency,^2^ in which the lead author or the manuscript guarantor will need to confirm that the manuscript is an honest account of the study being reported.

## 3 DATA SHARING

Data sharing, such that key elements from a study are available to peers for examination, replication, and validation, is an important aspect of reproducibility and transparency, and it is being mandated by an increasing number of funders.

As stated in Wiley's data sharing policy, “the sharing of data enables others to reuse experimental results and supports the creation of new work built on previous findings, improving the efficiencies of the research process, and supporting the critical goals of transparency and reproducibility.” Indeed, we agree that such “open research,” if possible in practice, facilitates faster and more effective research discovery.

For all manuscripts accepted on or after April 1, 2019, we will require a data sharing statement. Authors will need to state whether the data and materials used to conduct the research will be made available to any researcher for purposes of reproducing the results or replicating the procedures. In practical terms, authors will need to indicate whether the data and materials are available to the community, and, if so, how and where to access them. Such data should follow proper ethical guidelines and legal requirements for sharing, protecting participants' privacy. For data that cannot be shared, a short description of the restriction will need to be provided. If no data is associated with the article or all data underlying the results presented are available in the article, this will also need to be stated.

In addition, and following ICMJE guidelines, manuscripts submitted to the journal that report the results of clinical trials must contain a detailed data sharing statement, as described in Taichman et al.^3^ Further, clinical trials that begin enrolling participants on or after January 1, 2019, must include a data sharing plan in the trial's registration. While these statements will be required, they do not yet mandate data sharing. We will monitor how these requirements evolve in the community.

We hope that these initiatives, some of which are encompassed under the “Transparency and Openness Promotion” (TOP) guidelines (https://cos.io/top)—to which we adhere—will help us improve the transparency and reproducibility of published articles, and we will continue to work with you, the community, to enhance the quality and trustworthiness of medical research. We encourage authors to submit replication studies to *Health Science Reports*, particularly those attempting to reproduce findings reported in the journal, as such continued discussion is crucial for the advancement of science. Similarly, we welcome study protocols, which increase transparency in the design, conduct, and reporting of the resulting research articles.

## CONFLICTS OF INTEREST

The authors have declared that there is no conflict of interest.
